# Supplementary material for: Single cell immunophenotyping identifies CD8+ GZMK+ IFNG+ T cells as a key immune population in cutaneous Lyme disease
Source: bioRxiv. 2025 Jun 12:2025.06.09.658661. Preprint. [Version 1] doi: 10.1101/2025.06.09.658661 (PMC12259044; doi:10.1101/2025.06.09.658661)
Supplement: 1 [file NIHPP-2025.06.09.658661V1-supplement-1.pdf]

bioRxiv preprint doi: https://doi.org/10.1101/2025.06.09.658661; this version posted June 12, 2025. The copyright holder for this preprint (which was not certified by peer review) is the author/funder, who has granted bioRxiv a license to display the preprint in perpetuity. It is made available under aCC-BY 4.0 International license.

Supplemental Figures

Supplemental Figure 1

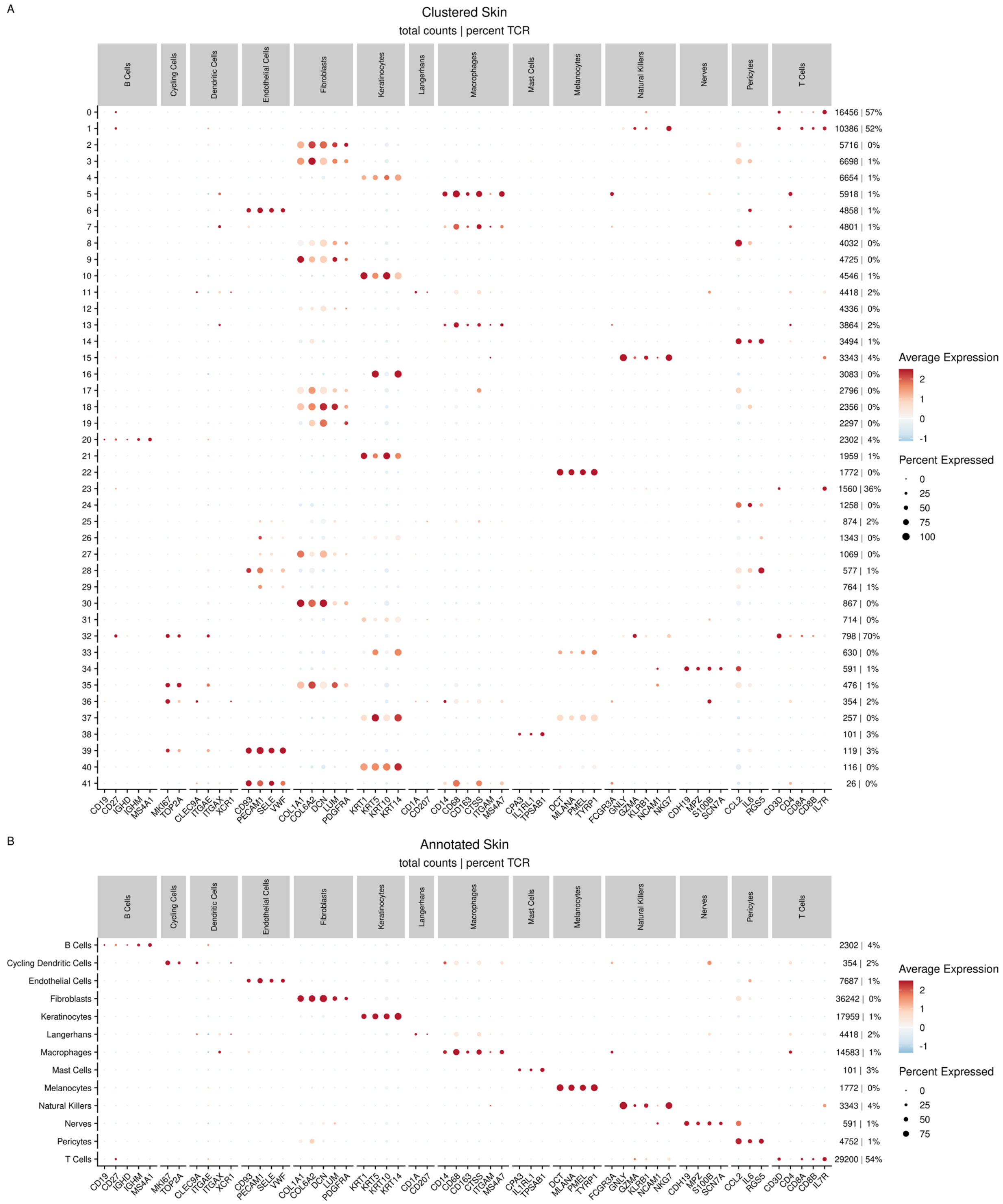

bioRxiv preprint doi: https://doi.org/10.1101/2025.06.09.658661; this version posted June 12, 2025. The copyright holder for this preprint (which was not certified by peer review) is the author/funder, who has granted bioRxiv a license to display the preprint in perpetuity. It is made available under aCC-BY 4.0 International license.

Supplemental Figure 1. Clustering and annotation of the skin. A) Dot plot showing the average expression of characteristic marker genes for each cluster within the skin. **B)** Dot plot showing the average expression of the same set of markers for each annotated cell type within the skin. Red indicates higher levels of expression and blue indicates lower levels of expression, with the size of the dot reflecting the percentage of that cluster of cell type that is expressing the corresponding marker. Numbers in the two columns on the right side of the plots indicate the total number of cells in that cluster or cell type and the percentage that have a paired TCR, respectively.

Supplemental Figure 2

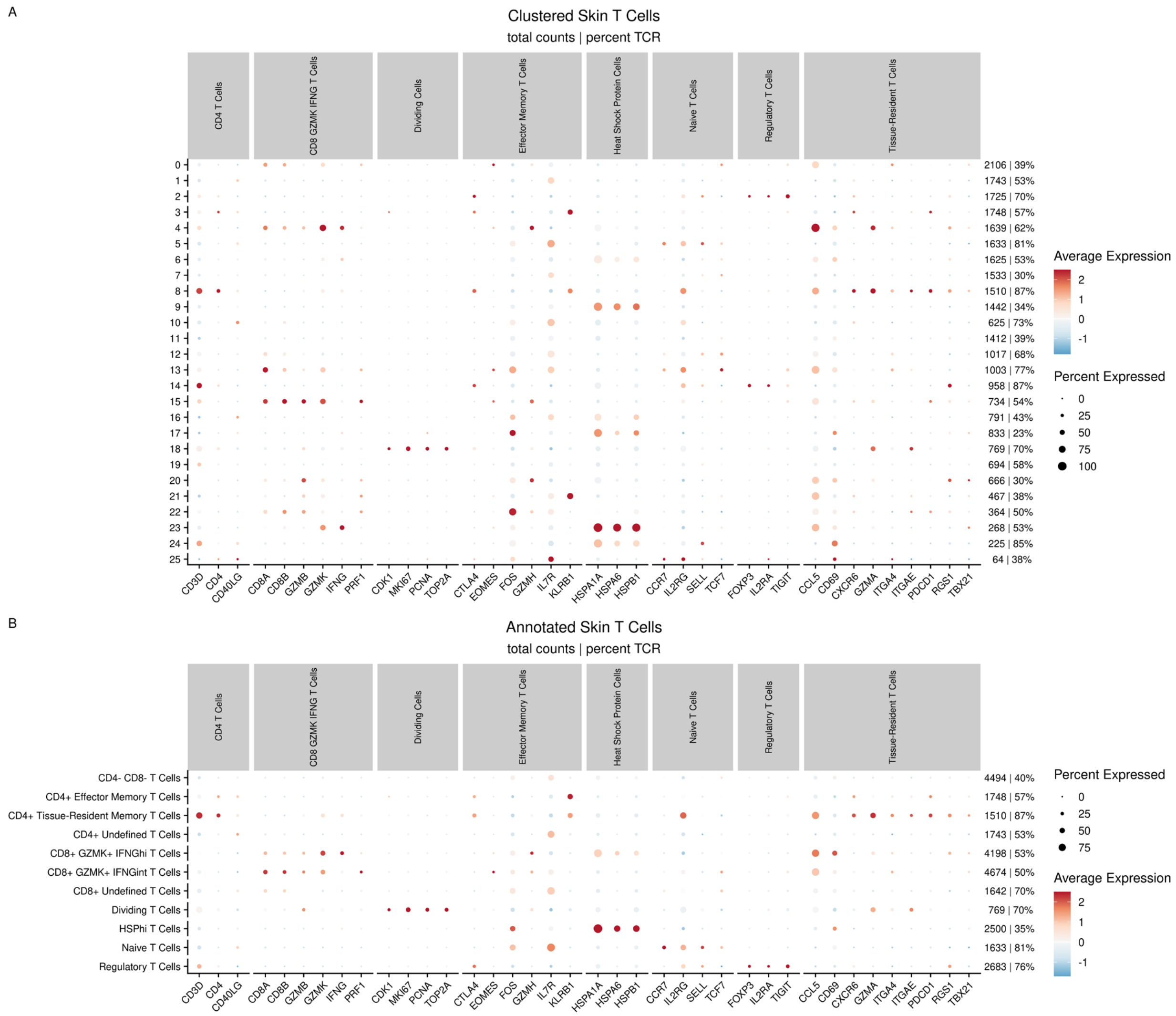

bioRxiv preprint doi: https://doi.org/10.1101/2025.06.09.658661; this version posted June 12, 2025. The copyright holder for this preprint (which was not certified by peer review) is the author/funder, who has granted bioRxiv a license to display the preprint in perpetuity. It is made available under aCC-BY 4.0 International license.

Supplemental Figure 2. Clustering and annotation of the skin T cells. A) Dot plot showing the average expression of characteristic marker genes for each cluster within the skin T cells. **B)** Dot plot showing the average expression of the same set of markers for each annotated cell type within the skin. Red indicates higher levels of expression and blue indicates lower levels of expression, with the size of the dot reflecting the percentage of that cluster of cell type which is expressing the corresponding marker. The two columns of information on the right side of the plots indicate the total number of cells in that cluster or cell type and the percentage which have a paired TCR respectively.

bioRxiv preprint doi: https://doi.org/10.1101/2025.06.09.658661; this version posted June 12, 2025. The copyright holder for this preprint (which was not certified by peer review) is the author/funder, who has granted bioRxiv a license to display the preprint in perpetuity. It is made available under aCC-BY 4.0 International license.

Supplemental Figure 3

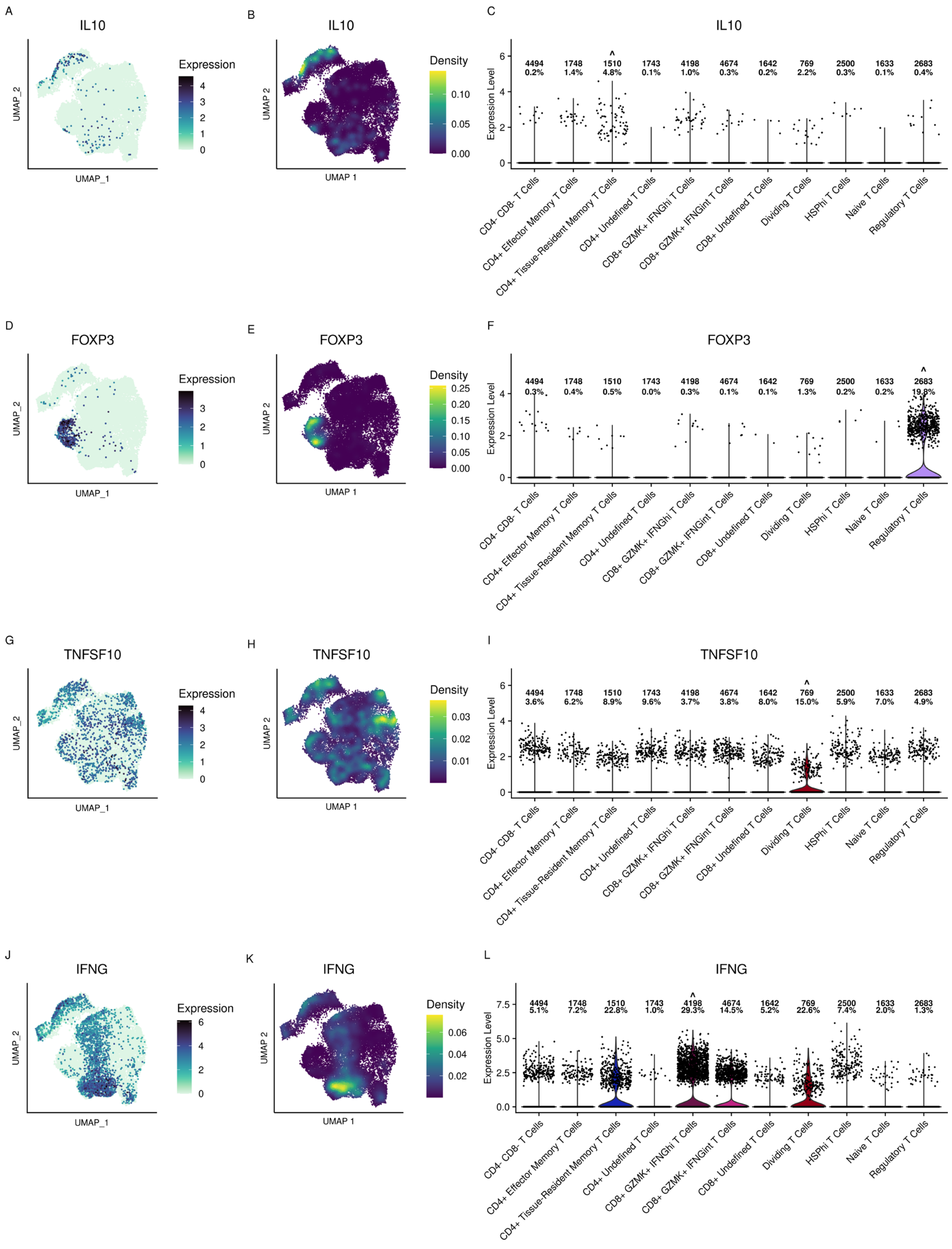

bioRxiv preprint doi: https://doi.org/10.1101/2025.06.09.658661; this version posted June 12, 2025. The copyright holder for this preprint (which was not certified by peer review) is the author/funder, who has granted bioRxiv a license to display the preprint in perpetuity. It is made available under aCC-BY 4.0 International license.

Supplemental Figure 3. Expression of select genes within the skin T cells. Each row shows the expression of IL10 (**A-C**), FOXP3 (**D-F**), TNFSF10/TRAIL (**G-I**), and IFNG (**J-L**) respectively. **A)**, **D)**, **G)**, and **J)** show the average expression of each gene upon the skin T cells UMAP, with darker colors corresponding to higher expression. **B)**, **E)**, **H)**, and **L)** show expression as a feature of kernel density using the Nebulosa package to allow for better visualization. **C)**, **F)**, **H)**, and **L)** show expression on a cell type level, with points representing individual cells. The top row of numbers within the violin plots is a count of how many cells are in that cell type, and the second row is the percentage of them with positive expression of the relative gene.

bioRxiv preprint doi: https://doi.org/10.1101/2025.06.09.658661; this version posted June 12, 2025. The copyright holder for this preprint (which was not certified by peer review) is the author/funder, who has granted bioRxiv a license to display the preprint in perpetuity. It is made available under aCC-BY 4.0 International license.

Supplemental Figure 4

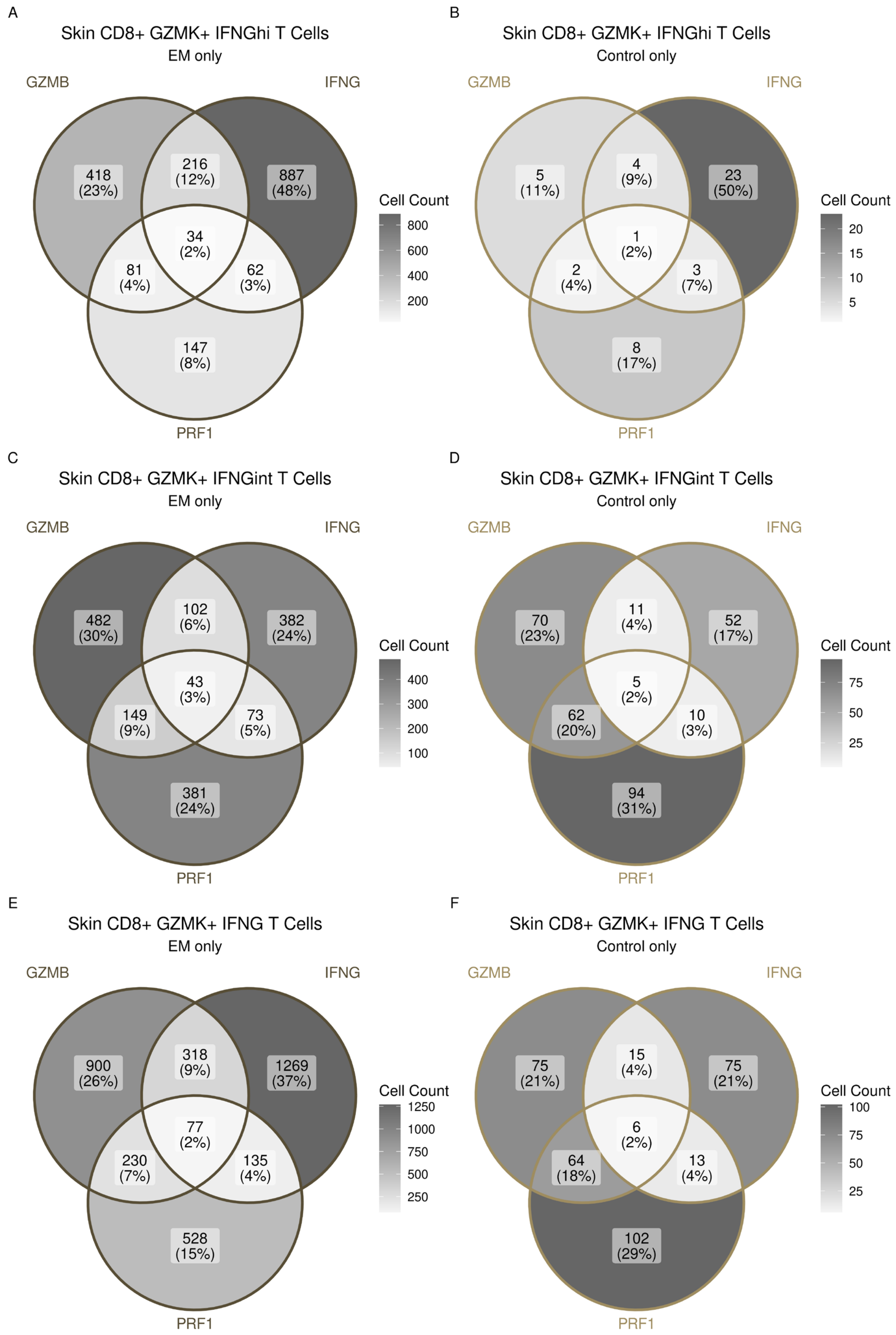

bioRxiv preprint doi: https://doi.org/10.1101/2025.06.09.658661; this version posted June 12, 2025. The copyright holder for this preprint (which was not certified by peer review) is the author/funder, who has granted bioRxiv a license to display the preprint in perpetuity. It is made available under aCC-BY 4.0 International license.

Supplemental Figure 4. Positive expression overlaps of GZMB, IFNG, and PRF1 within the CD8+ GZMK+ IFNG+ T cells. Each Venn diagram represents the number of cells with positive expression of GZMB, IFNG, and/or PRF1, with percentages relative to the total cells being represented in that Venn diagram. The left column is for only the EM subset and the right column is for only the control subset. **A)** and **B)** are for the CD8+ GZMK+ IFNGhi T cells, **C)** and **D)** are for the CD8+ GZMK+ IFNGint T cells, and **E)** and **F)** are for both cell types combined.

Supplemental Figure 5

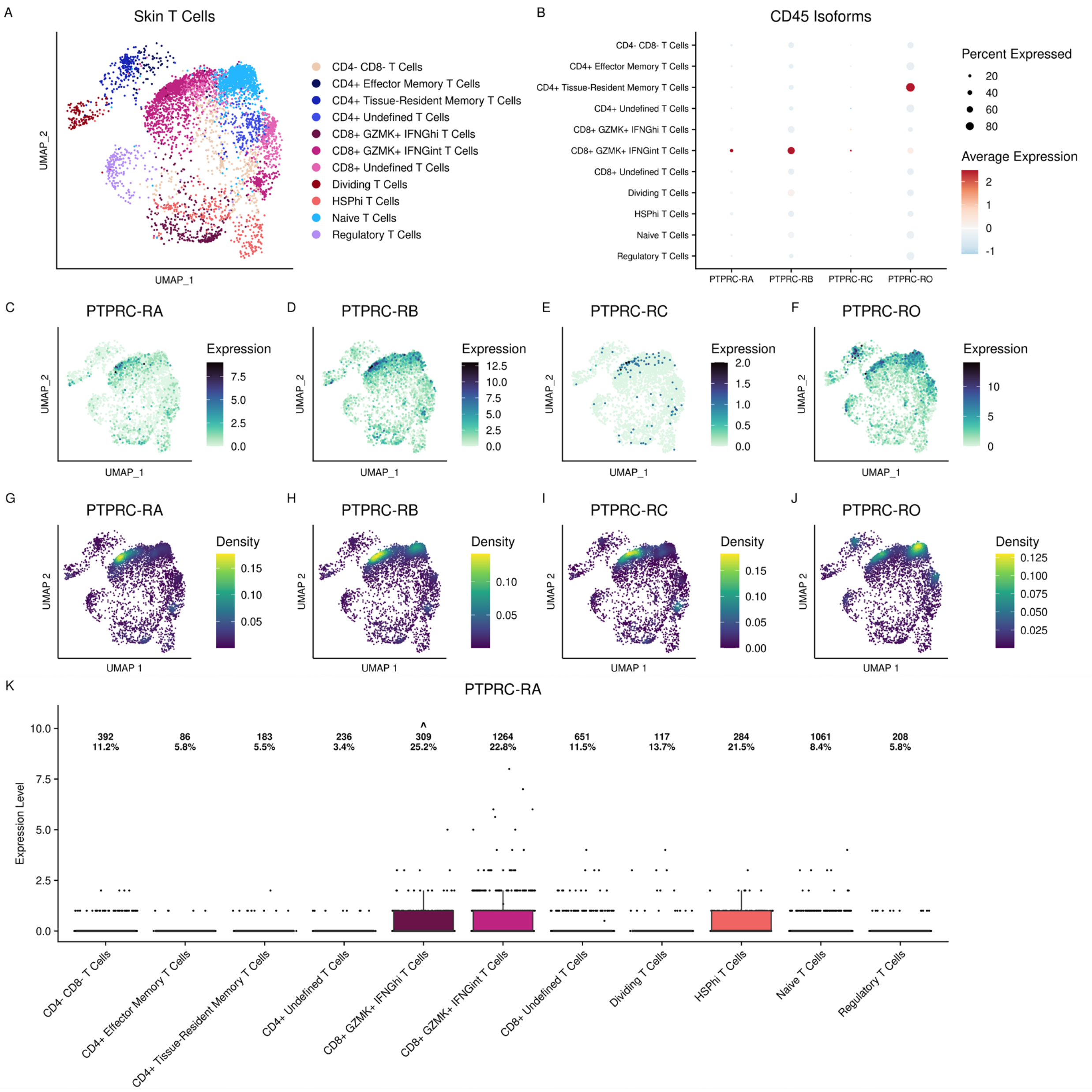

bioRxiv preprint doi: https://doi.org/10.1101/2025.06.09.658661; this version posted June 12, 2025. The copyright holder for this preprint (which was not certified by peer review) is the author/funder, who has granted bioRxiv a license to display the preprint in perpetuity. It is made available under aCC-BY 4.0 International license.

Supplemental Figure 5. CD45 spliced isoforms in skin T cells using IDEIS. A) UMAP showing all of the skin T cells that had a spliced isoform of CD45 (RA, RB, RC, or RO) detected with the IDEIS package. **B)** Dot plot showing the expression of each isoform per T cell subtype. **C-F)** Expression of each isoform upon the skin T cells UMAP, with darker colors corresponding to higher expression. **G-L)** Expression as a feature of kernel density using the Nebulosa package to allow for better visualization. **K)** Expression on a cell type level, with points representing individual cells. The top row of numbers within the box plot is a count of how many cells are in that cell type, and the second row is the percentage of them with positive expression of CD45RA.

Supplemental Figure 6

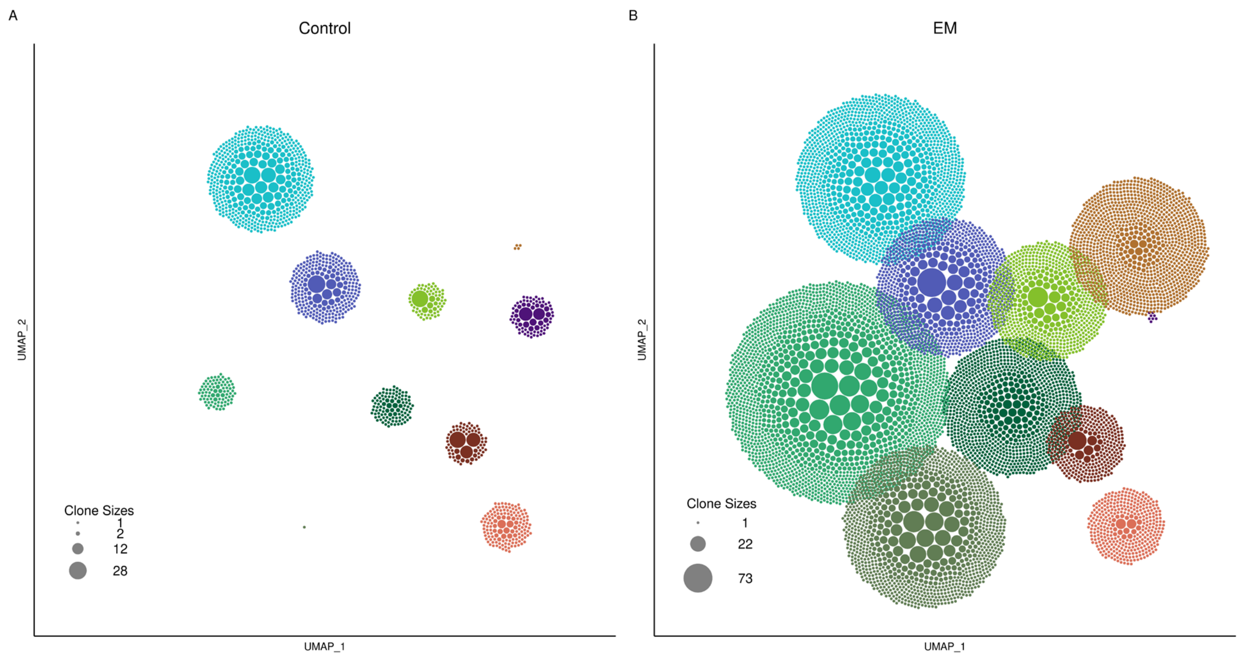

Supplemental Figure 6. Clonal expansion of skin T cells. A) and **B)** UMAP of the skin T cells in control and EM, respectively, with points representing clonotype sizes within each participant. The scale of the point size is consistent for both sample types. Participants without a paired control and EM are not shown.

bioRxiv preprint doi: https://doi.org/10.1101/2025.06.09.658661; this version posted June 12, 2025. The copyright holder for this preprint (which was not certified by peer review) is the author/funder, who has granted bioRxiv a license to display the preprint in perpetuity. It is made available under aCC-BY 4.0 International license.

Supplemental Figure 7

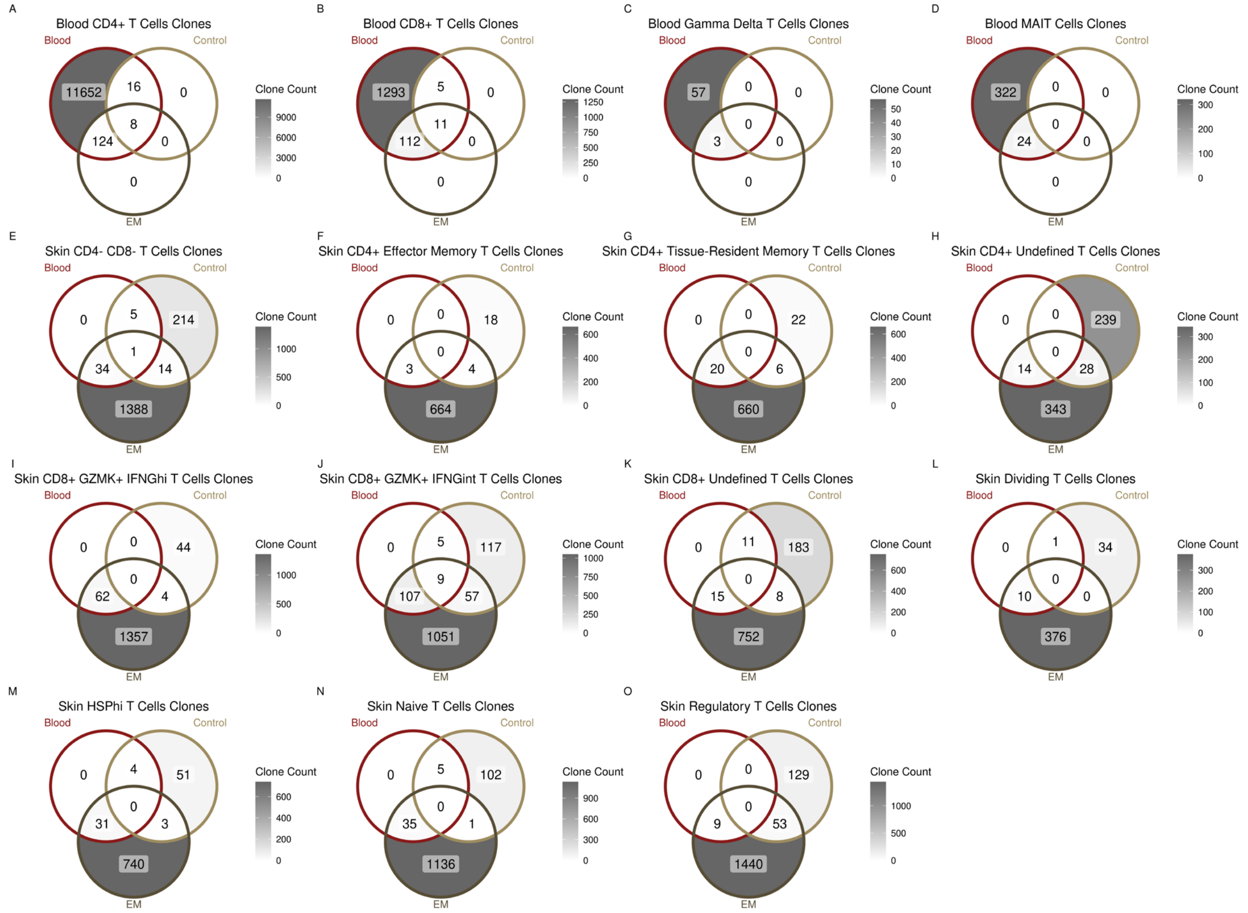

Supplemental Figure 7. T cell clonal overlaps between the blood and the skin. A-D) Clonal overlaps between 4 major blood T cell subtypes (CD4$^+$, CD8$^+$, $\gamma\delta$ T cells, and MAIT cells) and clonotypes within the EM and control skin samples. **E-O)** Clonotypes identified within each of the 11 defined T cell subtypes in EM and control skin samples that shared TCRs with blood samples. Numbers represent the count of unique TCRs identified and percentages represent the fraction of each segment compared to the total number of clonotypes within each respective Venn diagram.

bioRxiv preprint doi: https://doi.org/10.1101/2025.06.09.658661; this version posted June 12, 2025. The copyright holder for this preprint (which was not certified by peer review) is the author/funder, who has granted bioRxiv a license to display the preprint in perpetuity. It is made available under aCC-BY 4.0 International license.

Supplemental Figure 8

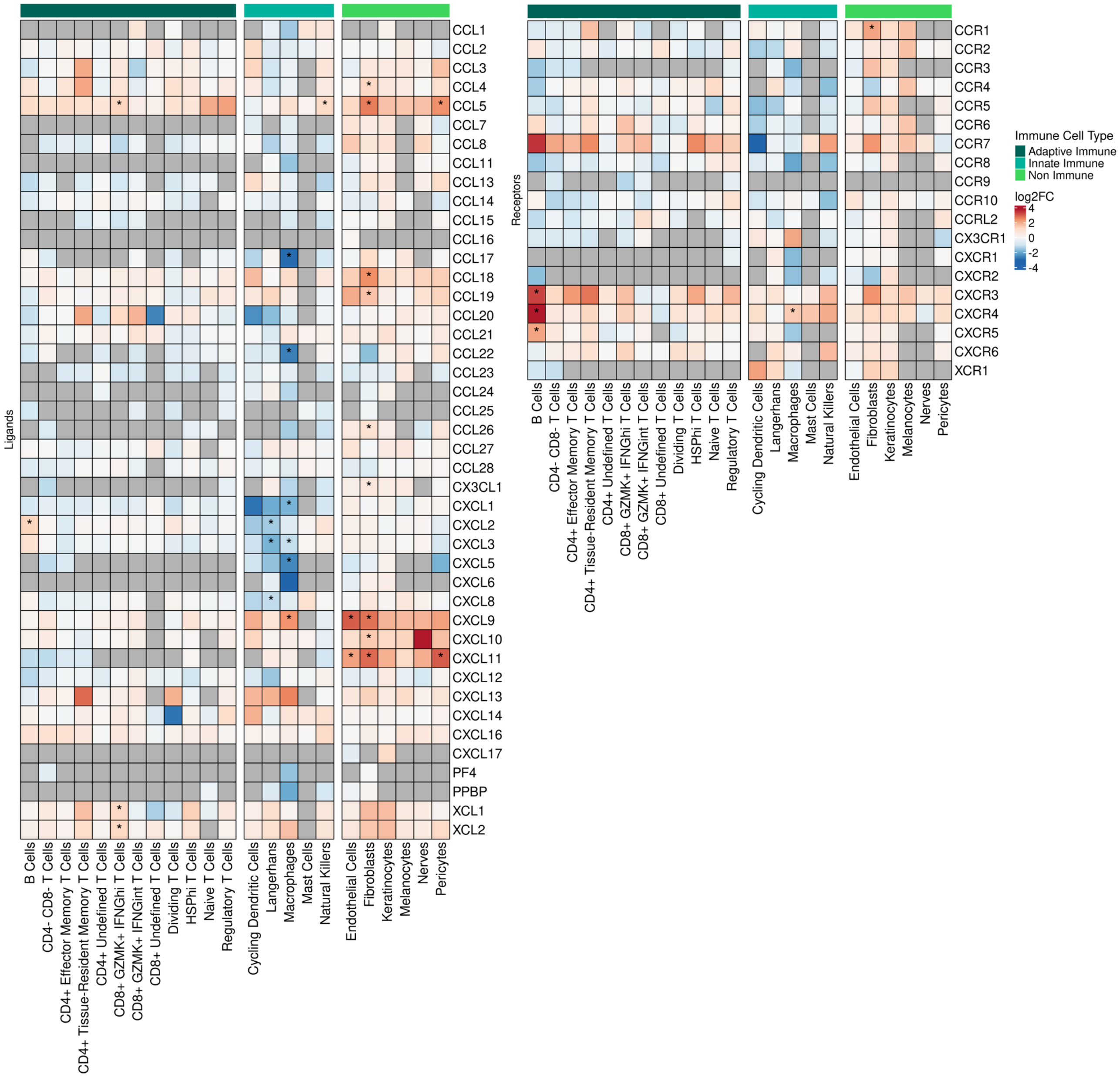

Supplemental Figure 8. Gene expression of chemokine ligand and receptor genes in the skin. Heatmap showing the average fold change of the expression of all chemokine-related genes across the full set of skin cell types. The bar on the top indicates whether or not the cell types being plotted are immune-related (dark green for adaptive immune cell types, cyan for innate immune cell types, and bright green for non-immune cell types). Within the heatmap itself, red indicates upregulation, and blue indicates downregulation, with asterisks denoting statistically significant changes (FDR < 0.05). Gray cells indicate no expression of the relevant gene for that

bioRxiv preprint doi: https://doi.org/10.1101/2025.06.09.658661; this version posted June 12, 2025. The copyright holder for this preprint (which was not certified by peer review) is the author/funder, who has granted bioRxiv a license to display the preprint in perpetuity. It is made available under aCC-BY 4.0 International license.

cell type (not applicable). Differential expression was calculated between the EM and control portions of each cell type. Note that PF4 is CXCL4 and PPBP is CXCL7.

Supplemental Figure 9

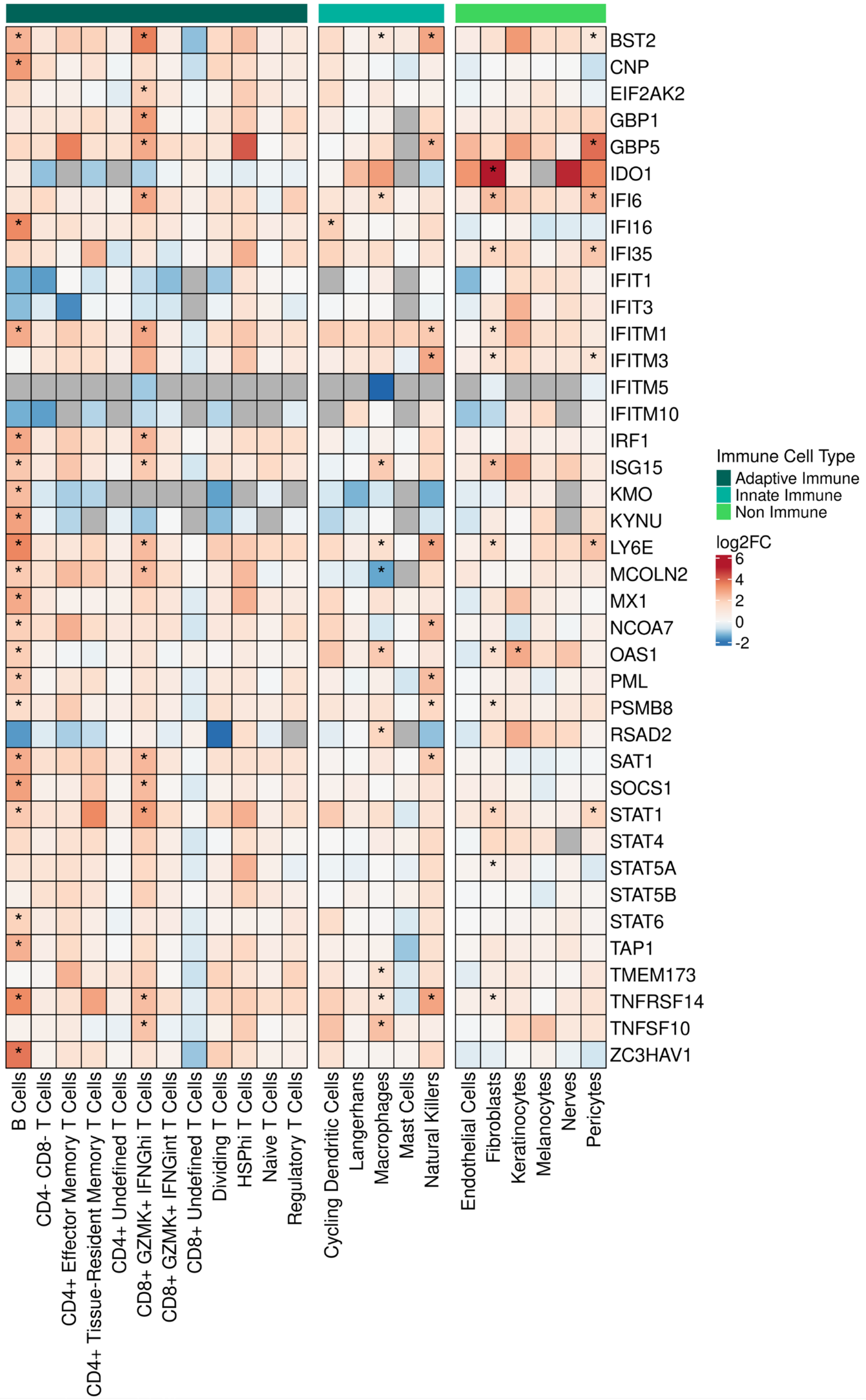

bioRxiv preprint doi: https://doi.org/10.1101/2025.06.09.658661; this version posted June 12, 2025. The copyright holder for this preprint (which was not certified by peer review) is the author/funder, who has granted bioRxiv a license to display the preprint in perpetuity. It is made available under aCC-BY 4.0 International license.

Supplemental Figure 9. Gene expression of interferon, interferon-regulated, interleukin, and signaling-related genes in the skin. Heatmap showing the average fold change of the expression of select genes across the full set of skin cell types. The bar on the top indicates whether or not the cell types being plotted are immune-related (dark green for adaptive immune cell types, cyan for innate immune cell types, and bright green for non-immune cell types). Within the heatmap itself, red indicates upregulation, and blue indicates downregulation, with asterisks denoting statistically significant changes (FDR < 0.05). Gray cells indicate no expression of the relevant gene for that cell type (not applicable). Differential expression was calculated between the EM and control portions of each cell type.

bioRxiv preprint doi: https://doi.org/10.1101/2025.06.09.658661; this version posted June 12, 2025. The copyright holder for this preprint (which was not certified by peer review) is the author/funder, who has granted bioRxiv a license to display the preprint in perpetuity. It is made available under aCC-BY 4.0 International license.

Supplemental Figure 10

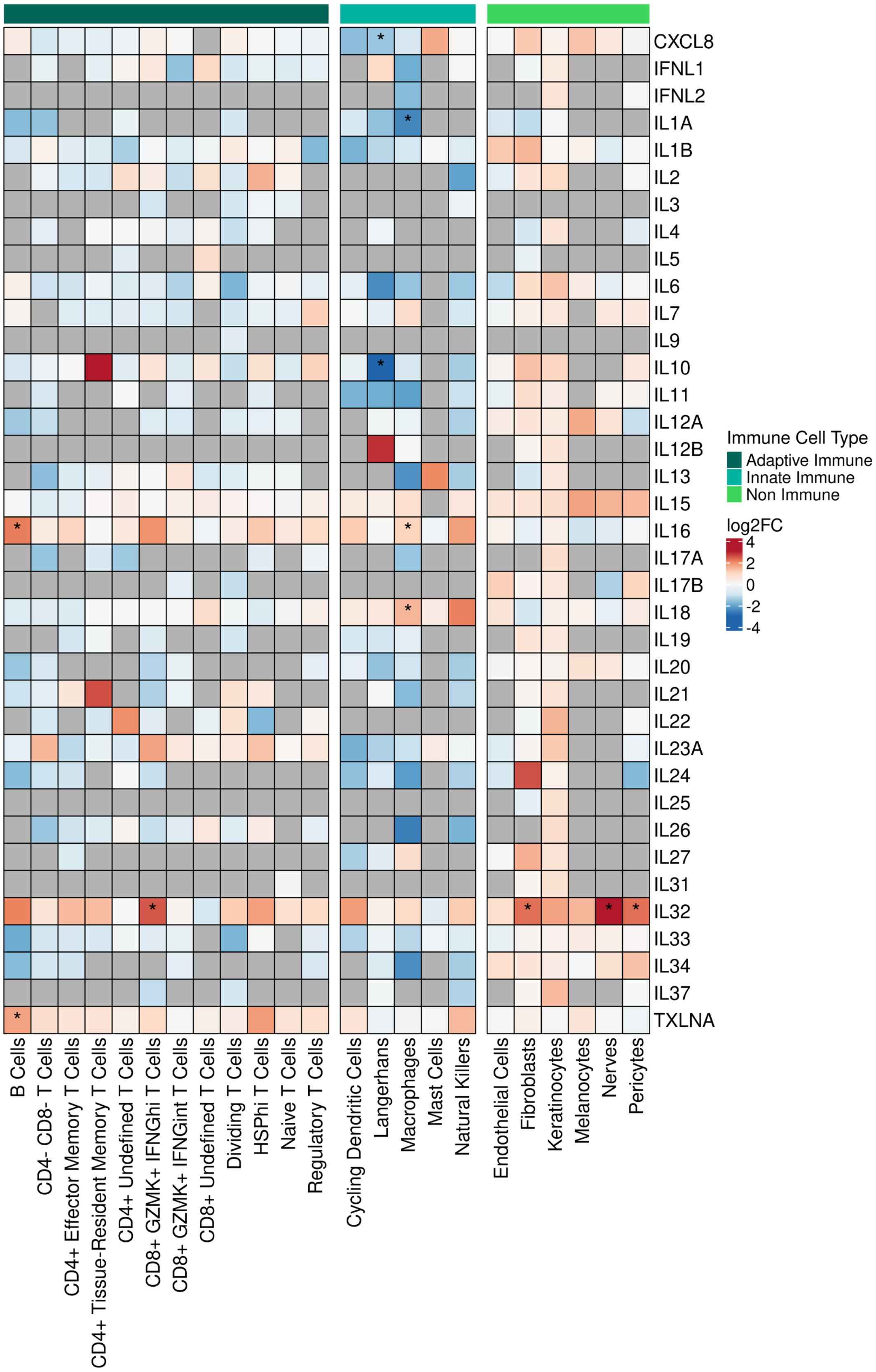

bioRxiv preprint doi: https://doi.org/10.1101/2025.06.09.658661; this version posted June 12, 2025. The copyright holder for this preprint (which was not certified by peer review) is the author/funder, who has granted bioRxiv a license to display the preprint in perpetuity. It is made available under aCC-BY 4.0 International license.

Supplemental Figure 10. Gene expression of interleukin ligand and receptor genes in the skin. Heatmap showing the average fold change of the expression of select interleukin genes across the full set of skin cell types. The bar on the top indicates whether or not the cell types being plotted are immune-related (dark green for adaptive immune cell types, cyan for innate immune cell types, and bright green for non-immune cell types). Within the heatmap itself, red indicates upregulation, and blue indicates downregulation, with asterisks denoting statistically significant changes (FDR < 0.05). Gray cells indicate no expression of the relevant gene for that cell type (not applicable). Differential expression was calculated between the EM and control portions of each cell type. Note that CXCL8 is IL8, TXLNA is IL14, IFNL1 is IL29, and IFNL2 is IL28A.

Supplemental Figure 11

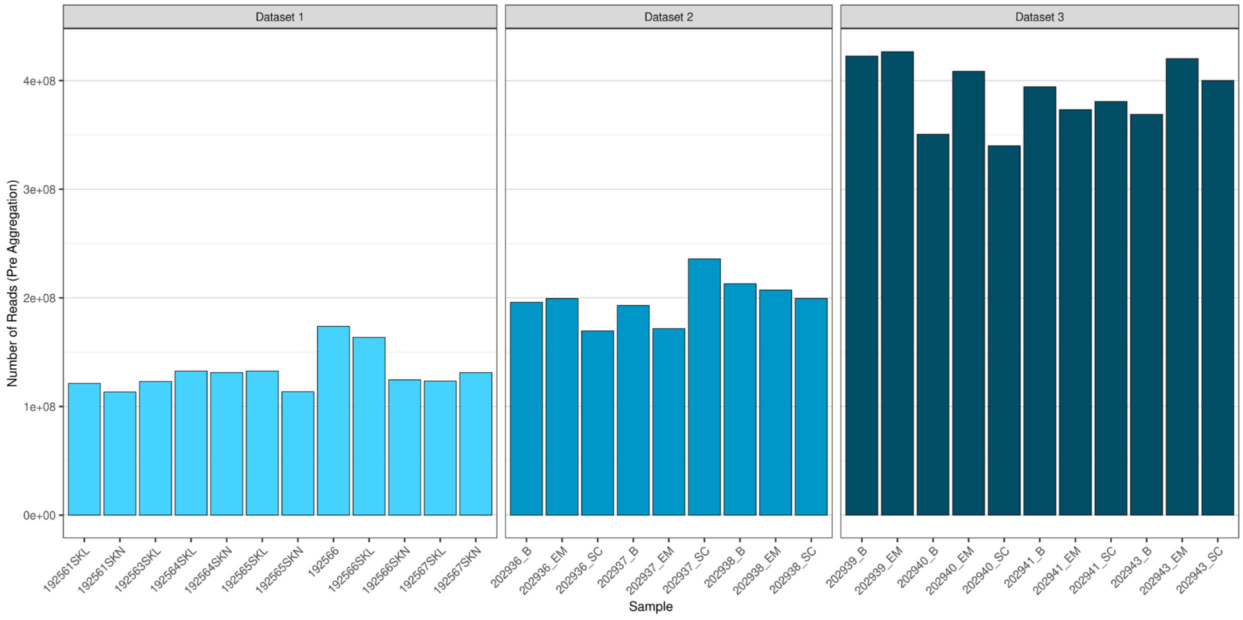

Supplemental Figure 11. Number of reads per sample before aggregation. Bar plot showing the number of reads in the hundreds of millions for each sample (blood and skin, including control and EM) prior to aggregation, grouped and colored by dataset.

bioRxiv preprint doi: https://doi.org/10.1101/2025.06.09.658661; this version posted June 12, 2025. The copyright holder for this preprint (which was not certified by peer review) is the author/funder, who has granted bioRxiv a license to display the preprint in perpetuity. It is made available under aCC-BY 4.0 International license.

Supplemental Figure 12

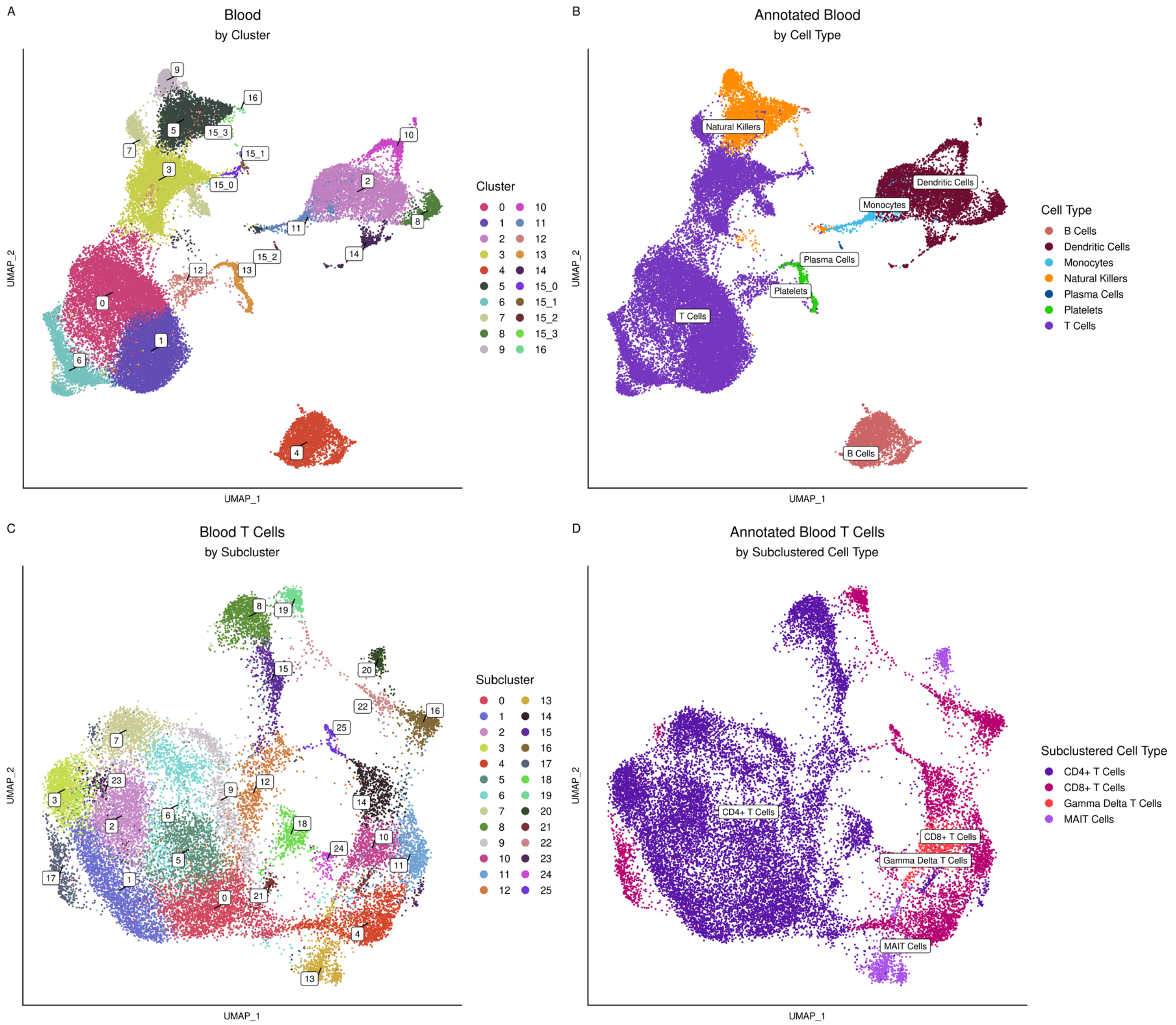

Supplemental Figure 12. Clustering and annotation of the blood and the blood T cells. A) UMAP showing the Seurat clusters for the blood data. Note that cluster 15 was split into four subclusters for annotation purposes. **B)** UMAP showing the annotated blood cell types. **C)** UMAP showing the Seurat clusters for the blood T cells. **D)** UMAP showing the annotated blood T cell types.
